# Radiologist Self-training: a Study of Cancer Detection when Reading Mammograms at Work Clinics or Workshops

**DOI:** 10.1007/s13187-022-02156-w

**Published:** 2022-05-05

**Authors:** Sarah J. Lewis, Natacha Borecky, Tong Li, Melissa L. Barron, Patrick Brennan, Phuong Dung Yun Trieu

**Affiliations:** 1 Discipline of Medical Imaging Science, Faculty of Medicine and Health, University of Sydney, D18 – Level 7 Susan Wakil Health Building, New South Wales 2006 Sydney, Australia; 2 BreastScreen New South Wales, Sydney, Australia

**Keywords:** Digital mammograms, Simulation, Remote training, Radiology, Breast cancer

## Abstract

Provision of online and remote specialist education and general continued professional education in medicine is a growing field. For radiology specifically, the ability to access web-based platforms that house high resolution medical images, and the high fidelity of simulated activities is increasingly growing due to positive changes in technology. This study investigates the differences in providing a self-directed specialist radiology education system in two modes: at clinics and in-person workshops. 335 Australian radiologists completed 562 readings of mammogram test sets through the web-based interactive BREAST platform with 325 at conference workshops and 237 at their workplaces. They engaged with test sets with each comprising of 60 mammogram cases (20 cancer and 40 normal). Radiologists marked the location of any cancers and had their performance measured via 5 metrics of diagnostic accuracy. Results show that the location of engagement with BREAST did not yield any significant difference in the performances of all radiologists and the same radiologists between two reading modes (*P* > 0.05). Radiologists who read screening mammograms for BreastScreen Australia performed better when they completed the test sets at designated workshops (*P* < 0.05), as was also the case for radiologists who read > 100 cases per week (*P* < 0.05). In contrast, radiologists who read less mammograms frequently recorded better performances in specificity and JAFROC at clinics (*P* < 0.05). Findings show that remotely accessed online education for specialised training and core skills building in radiology can provide a similar learning opportunity for breast radiologists when compared to on-site dedicated workshops at scientific meetings. For readers with high volumes of mammograms, a workshop setting may provide a superior experience while clinic setting is more helpful to less experienced readers.

## Introduction

Simulation in specialised areas of medicine, such as radiology, can be used as a continuing professional development (CPD) activity. High-fidelity simulation can allow health students and practitioners to engage in deliberate practice of specialised skills that imitate the clinical environment and build workforce capacity. Accessing simulation remotely has been popular in many educational arenas to assist with skills that would traditionally be undertaken in a laboratory, but can also be offered through on-line engagement systems such as Remote Labs (LabShare) or through dedicated platforms where users would acquire a subscription or access to a Learning Management System (LMS) [1]. Considering the large geographical area of Australia, there is a need to explore how specialised medical education can be provided to regional and rural practitioners [2]. Additionally, the global pandemic from the novel coronavirus (Covid-19) has seen most scientific gathering transition to virtual delivery, including associated workshops. Thus, it is important that online activities can be provided at equal quality and opportunity as face-to-face experiences.

Teleradiology, the interpretation of medical images by radiologists who might not be on-site at the same location as the patient has entered the mainstay of radiologists’ workflow internationally in the last 20 years. DICOM (Digital Imaging and Communications in Medicine) standard has allowed for standardisation of image data storage, printing, transmission through standard file formats and network communications coupled with PACS (Picture Archiving and Communications System) — the archiving and accessing of medical image sets; therefore, radiologists and other health practitioners are able to view DICOM images from a variety of locations [3]. The building blocks to access simulated and real patient images exist readily for most radiologists, and these include access to training sets of images. This paper focusses on CPD and education for radiologists who read mammograms for the purpose of cancer detection in Australia. The national standard for quality and accreditation is for radiologists to read at least 2000 cases per year, as number of cases read annually has strong links to higher performance [3]. For doctors to maintain their registration and accreditation, CPD is mandated in Australia [4].

The aim of this work is to investigate if on-line, remote access of high-fidelity educational CPD provides an equal educational opportunity to facilitated workshops in peer scientific gatherings.

## Methods

BREAST (the Breastscreen REader Assessment STrategy) (https://breast-australia.sydney.edu.au/) was developed in 2011 to provide an educational platform for CPD and core-skills training for radiologists. BREAST releases new yearly test sets, each comprising of 60 FFDM (Full-Field Digital Mammography) cases which radiologists read and mark the location of the cancer via an interactive web interface. BREAST has been provided through two mediums: through dedicated on-site workshops appended to national or state scientific meetings (radiology or breast multidisciplinary) or accessed via an on-line educational activity. Radiologists are able to undertake the training test set, which generally takes about 2 h, at their workplace provided they were able to access a PACS and high-resolution monitors. BREAST has been provided free to radiologists working for BreastScreen Australia (BSA) and at a nominal cost recovery to those who do not and is currently the recommended CPD activity by BSA and the Royal Australian and New Zealand College of Radiology (RANZCR) [4]. RANZCR CPD points are achieved through appellation of the BREAST program. New test sets are developed each year and research has shown that regular annual practice with BREAST can increase the diagnostic performance of radiologists by up to 30% [5]. Upon completion, radiologists are given a dashboard summary of their performance, indicating a range of matrices that show their ability to find cancers, recognise normal cases, and give specific location information (“ground truth”) about the site of the cancers.

Overarching ethics has been granted to collect data from BREAST radiologists (University of Sydney—Human Research Ethics Committee—2019/013). The de-identified mammograms of women who attended BSA are used in the construction of the test sets, ensuring real cases and a case mix represented of the Australian population including different types of cancer. Radiologists who enrolled with BREAST completed a self-reported questionnaire of their experience, case load, work type (BSA Reader or private reader) and are given a unique identifier code required for access.

### Test/Instrument Design

**Fig. 1:**
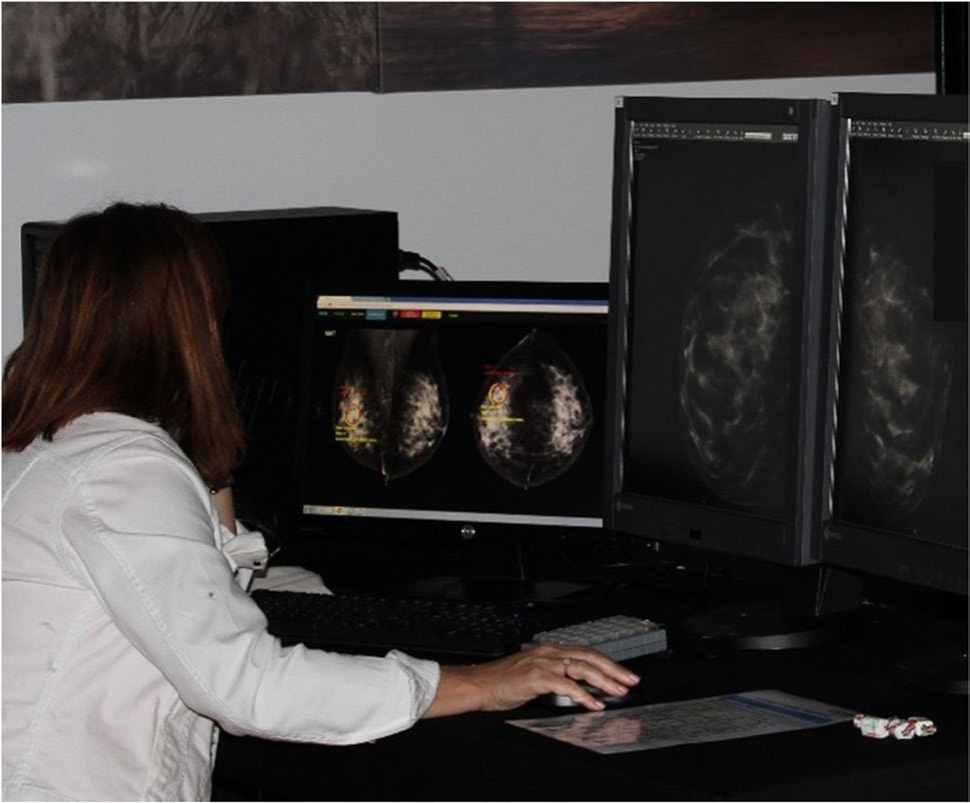
A radiologist was reading a mammogram BREAST test set

Each BREAST test set contains 60 cases with a third being cancer and set in a randomised order. Cancer cases used in training test sets were biopsy-proven while normal cases were confirmed by a negative 2-year following report. Radiologists are required to view each case and determined if the case requires a “recall” (there is suspicion of a cancer) or the case is normal, and the woman can “return to screen”. If the radiologist determines that a case was positive for cancer, they are instructed to place a Region of Interest (ROI) on the centre of the lesion and give a confidence rating between 2 and 5 that they consider the possibility of lesion being benign (rate 2) or cancerous (rate 3 to 5) in a similar system to the RANZCR reporting system [4]. The cases without any lesions detected are recorded as normal with rate 1. The display of mammograms is the same as would occur in a clinical environment, the main difference being that the test sets have a higher disease prevalence. At the conclusion of reading the test set, radiologists can scroll back through their decisions and access feedback about their performance, including their skills at determining cancer/no cancer, cancer location and cancer type. Figure 1 shows the activity setup.

### Facilitated Workshop versus Remote Access in the Clinic

Since 2011, BREAST has run a facilitated workshop (W) whereby radiologists booked into sessions to undertake a test set. At these dedicated workshops, reading conditions like the clinical environment are replicated including reduced ambient lighting. A SECTRA industry standard (PACS IDS7) is used to allow radiologists to access the cases in a standardised mammogram viewing format. The cases are viewed on high resolution monitors that meet BSA Quality Standards of 5 mega pixels or higher which are couriered to the venue. At most workshops, there are 4 dedicated facilitators to assist with questions and technology use and there are 4 separate workstations for bookings. Radiologists are generally instructed to complete the test set within the 2 h to allow for the next session to start on time.

Radiologists can also access BREAST test sets remotely which generally occurs in their workplaces (referred to in this paper as the clinic (C)). At the clinic, radiologists have access to the BREAST platform for the scoring software and BSA PACS to view DICOM images. Radiologists often email BREAST team for registration to BREAST platform and download instructions to access the mammogram test sets via their clinic PACS. There is no on-site facilitation from BREAST faculty and radiologists can undertake the same decision functions as workshop attendees as long as they have access to the high-resolution monitors. There are no time limits set for reading on the BREAST test set at the clinics, and some radiologist can choose to complete BREAST at any time they prefer.

### BREAST Feedback Mechanism

**Fig. 2:**
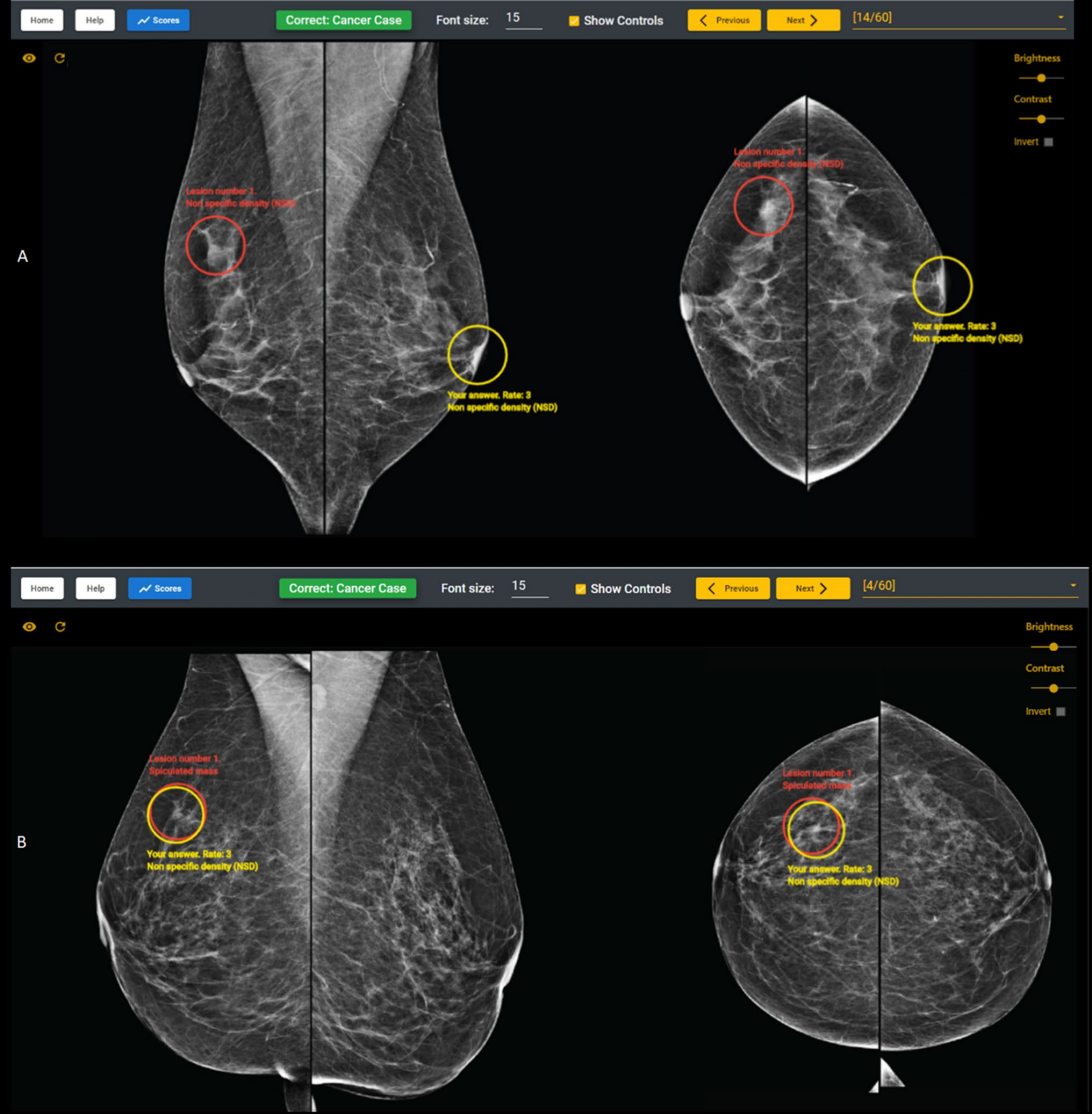
Feedback of a radiologist’ interpretation: **a**—Correct cancer case detection with an incorrect lesion location; **b**—Correct cancer case detection with correct lesion location

Table 1 provides an explanation of the performance data given to radiologists when they complete each BREAST test set. The highest order of performance is JAFROC (Jackknife alternative free-response receiver operating characteristic), which combines lesion sensitivity and specificity data, and gives an overall measure of a radiologist’s confidence to correct locate the site of a cancer while maintaining a high specificity and not recalling women unnecessarily while ROC (Receiver Operator Characteristic) is a combination of case sensitivity and specificity. Radiologists receive their feedback via a score calculation, giving information about their performance in relation to other radiologists and what the individual measures mean (sensitivity, specificity, lesion sensitivity, ROC and JAFROC). For each of the mammography cases, radiologists also receive feedback about how correct their location sensitivity decision was and the true location of any cancers (Fig. 2a and b).

**Table 1:** Reading performances of radiologists in different groups of experience between workshop (W) and clinic (C)

Role	Location (number of readings)	Specificity	Sensitivity	Lesion sensitivity	ROC	JAFROC
Non-BSA radiologists	Clinics (89)	0.793 ± 0.126	0.745 ± 0.180	0.635 ± 0.203	0.801 ± 0.103	0.683 ± 0.129
	Workshops (169)	0.733 ± 0.171	0.735 ± 0.169	0.592 ± 0.193	0.779 ± 0.093	0.626 ± 0.148
	P value	0.007	0.441	0.072	0.045	0.002
BSA radiologists	Clinics (148)	0.777 ± 0.139	0.782 ± 0.153	0.688 ± 0.176	0.819 ± 0.087	0.706 ± 0.118
	Workshops (156)	0.800 ± 0.144	0.845 ± 0.120	0.777 ± 0.131	0.867 ± 0.058	0.785 ± 0.083
	P value	0.036	< 0.0001	< 0.0001	< 0.0001	< 0.0001
Radiologists read ≤ 100 cases per week	Clinics (133)	0.79 ± 0.134	0.751 ± 0.168	0.643 ± 0.186	0.802 ± 0.094	0.685 ± 0.121
	Workshops (204)	0.738 ± 0.175	0.751 ± 0.172	0.618 ± 0.199	0.790 ± 0.093	0.649 ± 0.149
	P value	0.007	0.982	0.304	0.243	0.040
Radiologists read > 100 cases per week	Clinics (104)	0.774 ± 0.133	0.790 ± 0.158	0.688 ± 0.187	0.824 ± 0.092	0.712 ± 0.123
	Workshops (121)	0.811 ± 0.127	0.850 ± 0.102	0.786 ± 0.11	0.873 ± 0.051	0.792 ± 0.078
	P value	0.009	0.016	< 0.0001	< 0.0001	< 0.0001

### Data analysis

Data collected from the BREAST system was extracted between February 2013 and September 2021, including the radiologists’ performance and the location where they completed the BREAST test set — radiologists were then classified into a workshop (W) or clinic (C) group. The performance of readers with different experiences between two groups was compared in all metrics: sensitivity, specificity, lesion sensitivity, ROC AUC and JAFROC using the Mann–Whitney U test. The definition of metrics is described as below:

- Sensitivity: Ratio of the number of cancer cases a reader correctly identified (rate 3 to 5) versus the overall number of cancer cases
- Specificity: Percentage of negative case selections (rate 1 or 2) a reader made that corresponds to the true normal cases
- Lesion sensitivity: Ratio of the number of malignant lesions a reader correctly identified with rate 3, 4 or 5 versus the overall number of true lesions
- ROC AUC (Receiver Operating Characteristic Area Under Curve) [6]: The measure of the ability of a reader to distinguish between abnormal cases and normal cases through confidence ratings
- JAFROC (Jackknife Free-response Receiver Operating Characteristic) [7]: A method for measuring reader performance in lesion localization tasks in combination of identifying normal cases taking account of confidence ratings.

The paired comparison of the radiologists who owned the same working position and similar number of mammograms reading per week when they completed test sets at clinic and workshop was also included using the Wilcoxon signed ranked test.

With the data collection from 2019 onwards, the BREAST platform allowed for time measurements (Participant START and STOP) to be recorded, meaning it was possible to know the total maximum time the radiologists spent undertaking the test set of 60 cases. In this paper, we also conducted a brief comparison of time for radiologists that completed the test set in less than and more than 3 h, considering that time limit for each reading session at BREAST workshops while radiologists reading the mammogram test sets at the clinic were not limited in time for the test set completion.

Statistical tests were performed via SPSS software with* P* < 0.05 (2-tailed) considered as significant result.

## Results

There were 562 first-time readings of seven BREAST mammogram test sets performed by 335 Australian radiologists with 325 completed at workshops and 237 at clinics. The readers in the workshop group had an average of 12 years in reading mammograms while the ones in the clinic group was 10.5 years. The first-time reading of one test set by one reader was counted as one reading. Test sets had equal level of difficulty. Non-first-time readings (duplicated readings of test sets) were excluded in the analysis. Radiologists were also split into categories as either being employed in some capacity (full time or part time) as a reader for BSA or not at all. There was also separation of data around the industry benchmark of reading over 100 cases per week or under, which would provide some clarity around the performance of radiologists that read less cases, may work part time in private practices, or may work in more regional areas where the case loads are lower.

**Fig. 3:**
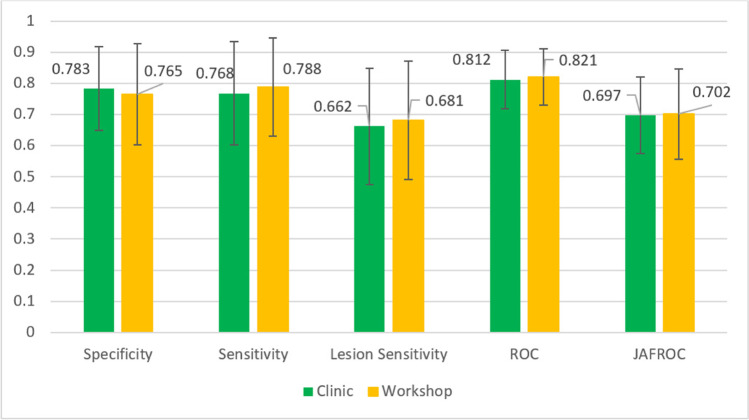
Overall performances of radiologists at Workshop (W) and Clinic (C)

When all participating radiologists were combined, there was no significant difference in diagnostic performance metrics between those that read BREAST test sets through a facilitated workshop (W) appended to a national scientific meeting and those that accessed the CPD tool at their workplace (C) (*P* > 0.05) (Fig. 3).

For 258 readings by radiologists who did not read for BSA and were therefore likely to be undertaking less FFDM screening cases loads but perhaps a reasonable amount of diagnostic/symptomatic work, their performance at clinics were significantly better than at workshops in specificity, ROC and JAFROC (*P* < 0.05). With 304 reading sessions completed by readers working for BSA, Table 1 clearly demonstrates that their performance for all measures was significantly higher when they engaged with BREAST at a workshop rather than at the clinic (*P* < 0.05).

In addition, the data shows that 325 readings at the workshop by radiologists who read less than 100 cases per week were significantly better in specificity and JAFROC compared with those done at the clinic (*P* < 0.05); however, 225 readings by radiologists who were heavy case readers (≥ 100 cases per week) were significantly better at a workshop than at clinic in all measures (*P* < 0.05) (Table 1).

When comparing the performances of BS and non-BS radiologists, no significant differences were found between these two groups when reading at clinics. However, higher diagnostic accuracy was reported by BS readers reading at workshops compared with non-BS readers in specificity, sensitivity, lesion sensitivity, ROC and JAFROC (*P* < 0.05) (Table 2).

**Table 2:** Comparison of mammogram reading performances of non-BSA and BSA radiologists in clinics and workshops

Reading locations	Role (number of readings)	Specificity	Sensitivity	Lesion Sensitivity	ROC	JAFROC
Clinics	Non-BSA (89)	0.793 ± 0.126	0.745 ± 0.18	0.635 ± 0.203	0.801 ± 0.103	0.683 ± 0.129
	BSA (148)	0.777 ± 0.139	0.782 ± 0.153	0.688 ± 0.176	0.819 ± 0.087	0.706 ± 0.118
	P value	0.454	0.147	0.101	0.315	0.240
Workshops	Non-BSA (169)	0.733 ± 0.171	0.735 ± 0.169	0.592 ± 0.193	0.779 ± 0.093	0.626 ± 0.148
	BSA (156)	0.800 ± 0.144	0.845 ± 0.12	0.777 ± 0.131	0.867 ± 0.058	0.785 ± 0.083
	P value	< 0.0001	< 0.0001	< 0.0001	< 0.0001	< 0.0001

With analysing the same radiologists who completed the BREAST test sets at both clinic and conference and had the same working status in both reading modes, there were 13 readers who worked for BreastScreen and read at least 60 cases per week. No significant differences in specificity, sensitivity, lesion sensitivity, ROC and JAFROC (*P* > 0.05) were found among these readers between two reading locations (Table 3).

**Table 3:** Paired comparison of reading performances of 13 BreastScreen radiologists at clinics and workshops

Location	Specificity	Sensitivity	Lesion Sensitivity	ROC	JAFROC
Clinics	0.730 ± 0.187	0.856 ± 0.09	0.770 ± 0.122	0.845 ± 0.065	0.745 ± 0.08
Workshops	0.810 ± 0.128	0.836 ± 0.094	0.760 ± 0.108	0.856 ± 0.054	0.772 ± 0.106
P value	0.093	0.322	0.583	0.552	0.279

Over 562 readings, 256 sessions had the completion time recorded. An analysis comparing the time taken to complete the BREAST test sets showed that 96% of radiologists (68 over 71 readings) completed their reading less than 3 h for each set while this rate at clinic was 65% (121 over 185 readings). Radiologists on average spent 86 min for each test set at the workshop and 82 min at the clinic with no significant difference between these reading modes (*P* = 0.135). There was also no significant difference in the time reading of BreastScreen radiologists at workshop (78 min) and at clinic (85 min) (P = 0.619).

## Discussion

The debate about the effectiveness of high-fidelity simulation, provided with facilitation at workshops or remote access, remains. In light of the Covid-19 pandemic, which has resulted in many cancelled educational gathering such as face to face workshops, conferences, scientific meetings and on-campus learning, the ability to provide meaningful CPD or core-skills education via remote access has become extremely important. Within health sciences, there is some research indicating that remote access assessment, when compared to face to face, is as effective for quantifying learning, such as with virtual skills assessments [1, 3]. However, there is limited published data on the provision of specialised medical CPD provided online, especially in the area of radiology, which lends itself to remote education due to the availability of storage, retrieval and transportation of DICOM images via PACS.

This paper, for the first time, provides data on a wide-scale learning activity for radiologists in Australia designed as either CPD or for core-skills learning. We report that remote access on-line learning and assessment for large numbers of tertiary referral specialists were just as effective as facilitated workshop testing. For most radiologists, the location and environment they accessed BREAST did not impact upon their performance scores, and they were able to engage in a highly technical CPD or core-skills development (depending on their stage of practice) from any geographical location in Australia. When we analysed the same breast screen readers who had at least 100 cases per week and completed the test sets at both clinic and conference, no significant difference in the readers’ performance metrics was found between two reading modes.

We found that remote delivery aided the performance of readers with low volume reading, but not for breast screen readers or readers with high volume of readings. For radiologists who are dedicated BSA readers, and/or who read over 100 cases per week, these participants performed the simulation task better when at a workshop. This may represent a scenario by which radiologists who attended conferences in general are more likely to take a vested interest in their extended learning, or it may be that a workshop promotes a friendly sense of competition. Additionally, many expert radiologists may be present at workshops as they are able to combine other professional representations such as committee work and giving presentations. However, the workshops do provide a generally quiet and disruption-free experience and this cannot be ruled out when considering performance.

From the opposite angle, radiologists that read equal to and fewer than 100 cases per week, and either have less mammography reading due to their geographical location, part time status and/or variety of case load across other radiology areas performed better in recognising normal cases (specificity), ROC and JAFROC when completing the BREAST test set at clinics. This finding was similar with a study conducting an online survey of student experience with remote exam delivery and compared test performance in remote versus in campus-based forms of similar assessments for medical students, and the results show that the remote delivery aided candidate performance in the Year 4 exam, but not in Year 5 [8]. With the remote online assessments, candidates could quickly access and appraise relevant information to support clinical decision-making which is now a vital part of modern medical practice, underpinning professional values and patient safety.

There is a limitation of this study which was a lack of data about environment factors that might impact on the performances of readers at the clinics, and this will require further investigation. In addition, while our results are encouraging about self-training and self-assessment through online system, it is difficult to clearly separate any effects arising specifically from lockdown.

## Conclusion

The overall performance of radiologists to engage with an accredited CPD/core skills educational tool was not related to the location of their learning. Radiologists’ reading performance via BREAST, considering the first time they engaged with the test set format did not depend on the mode of engagement, such as being remote access online or through facilitated workshops. However, BreastScreen readers or radiologists with high volume of reading did perform better at the workshops and in contrast, the non-BreastScreen readers or radiologists with low volume of reading seemed to perform better at clinics. The findings suggest that radiologists generally were able to navigate technologically advanced educational platforms without face-to-face instruction or peer interaction without impacting upon their performance. For the distinct cohorts of low volume readers, reading at clinics was better than at workshop while BreastScreen Australia readers and those readers who have a heavy mammography caseload, these participants performed better as a group at a facilitated workshop, likely a manifestation of their interest and expertise in breast imaging, education and research. With travel restrictions limiting physical networking during the Covid-19 pandemic and face to face learning opportunities, we show here using large numbers of medical specialists that high-fidelity simulation of radiology education using real cases curated into a test set and being accessed remotely provides excellent CPD opportunities.
